# Management of Acute Limb Ischaemia Due to COVID-19 Induced Arterial Thrombosis: A Multi-Centre Indian Experience

**DOI:** 10.3400/avd.oa.22-00012

**Published:** 2022-06-25

**Authors:** Natarajan Sekar, Jithin Jagan, Arunagiri Viruthagiri, Nedounsejiane Mandjiny, Karthikeyan Sivagnanam

**Affiliations:** 1 Kauvery Hospital, Chennai, India; 2 Kauvery Hospital, Tiruchirapalli, India; 3 Kauvery Hospital, Salem, India

**Keywords:** acute limb ischaemia, COVID-19, arterial thrombosis

## Abstract

**Objective:** To determine the outcomes following various surgical and medical treatments of Coronavirus disease 2019 (COVID-19) induced acute limb ischaemia.

**Methods:** A retrospective study of patients presenting with COVID induced arterial ischaemia in three hospitals from Southern India during the months of May 2020 to August 2021 was undertaken. These patients were managed by either thrombectomy, primary bypass, thrombolysis, anticoagulation or primary amputation based on the stage of ischaemia and the severity of COVID.

**Results:** A total of 67 limbs in 59 patients were analysed. The average time to intervention was 15 days. Upper limb involvement was seen in 16 and lower limb in 51 limbs. Of the 67 limbs, 39 (58.2%) were treated by open surgical revascularisation, 5 (7.4%) by catheter directed lysis, 17 (25.3%) were managed conservatively and 6 (8.9%) underwent primary amputation. Successful revascularisation could be carried out in 88.6% of patients. A limb salvage rate of 80.6% was achieved in these patients with a re-intervention rate of 13.6%. Major amputation rate was 14.92% and mortality was 13.56%.

**Conclusion:** Limb ischaemia after COVID can be safely managed by open thrombectomy or bypass. Similar rates of limb salvage as in non-COVID acute limb ischaemia can be obtained.

## Introduction

Coronavirus disease 2019 (COVID) caused by the severe acute respiratory syndrome corona virus-2 (SARS CoV-2) has been shown to be associated with vascular thrombosis. The virus causes an immune cross reaction with the endothelium leading to endothelial inflammation and an inflammatory occlusive thrombus.^1)^ During the first wave there was a sudden and significant increase of COVID infected patients presenting with acute limb ischaemia (ALI) noted at our institution. These cases seemed to occur in both asymptomatic and severe COVID patients alike and even weeks after discharge. Routine use of anticoagulation for inpatients as well as post-discharge brought down the incidence of ALI after COVID. Arterial thrombosis has been reported in 3.7% of patients with severe COVID despite prophylactic anticoagulation.^2)^ Arterial thromboembolic complications may have devastating consequences, including limb loss, premature intubation, multiorgan dysfunction, stroke, and death. Results of revascularisation for COVID induced limb ischaemia has been reported to be poor.^3)^ This study aims to describe the characteristics, clinical outcomes, pitfalls, lessons learnt and changes adopted in treating these patients presenting with arterial thrombosis due to COVID at three tertiary care hospitals in the state of Tamil Nadu in South India.

## Materials and Methods

With permission from the institutional ethics committee (Approval no: ECR/966/Inst/TN 2017/RR-21 dt 23rd October 2021), a retrospective observational study of patients presenting with acute arterial thrombosis due to COVID from May 2020 to August 2021 was carried out from three centres in the state of Tamil Nadu in South India. The data from all patients presenting with COVID induced acute limb ischaemia requiring urgent treatment were collected in a prospectively maintained database. These patients had tested positive for COVID either by reverse transcription polymerase chain reaction (RT-PCR) or COVID antibody (IGM) positivity coupled with computed tomography (CT) chest findings typical of COVID-19 (COVID-19 reporting and data system CO-RADS 5). None of these patients had received vaccination at the time of presentation. Asymptomatic patients were defined as those patients with no symptoms suggestive of COVID but had pulmonary CT features of COVID and were antibody (IGM) positive without having taken the vaccine. Mild cases were those patients who had symptoms of COVID and were treated medically without oxygen requirement. Majority of them were on home quarantine. Moderate COVID were patients who had shortness of breath requiring hospitalisation and oxygen therapy. Severe cases were defined as patients who required intensive care unit (ICU) care for shortness of breath with non-invasive or invasive ventilation. CT arteriogram was carried out for all patients and acute thrombosis was confirmed. COVID treatment was carried out as per the institutional protocol in these patients. All patients had hypercoagulability workup done to rule out thrombophilia. Those with thrombophilia were excluded. For all cases, echocardiography was performed to rule out a cardiac source of embolism.

### Procedure

Patients with acute limb ischaemia were classified based on Rutherford classification. CT aortogram and peripheral arteriogram was carried out for all patients. A bolus dose of heparin (100 U/kg) was administered on confirming arterial thrombosis. Patients were considered for open surgical thrombectomy or catheter directed lysis or conservative management based on time to presentation, Rutherford class of acute limb ischaemia, vessels involved and the severity of COVID. All procedures were carried out by one of five vascular surgeons operating in these three centres. Thrombectomy for upper limb was carried out by a trans-brachial route. Lower limb embolectomy was carried out either by a trans-femoral or trans-popliteal approach. Five patients needed both trans-femoral and distal trans-popliteal thrombectomy. Intra-operative angiogram was considered if the results were sub-optimal. Intra-operative instillation of 50,000–100,000 units of urokinase (U-FRAG, Bharat serums and vaccines limited, Mumbai, India) was given to the distal vascular bed in patients who underwent thrombectomy. Fasciotomy was carried out in patients with features suggestive of compartment syndrome or questionable muscle viability. Post-operatively all patients were maintained on an infusion of unfractionated heparin (UFH) and were titrated to maintain an activated partial thromboplastin time of 1.5–2 times the control for 48 hours. This was followed by subcutaneous low molecular weight heparin (LMWH) enoxaparin 1 mg per kg body weight, twice a day, till discharge. Some patients were given infusion of low molecular weight dextran (15 ml/h) for 24 hours. They were discharged either on rivaroxaban 15 mg twice a day for 3 weeks followed by 20 mg once a day or on warfarin with international normalised ratio maintained between 2.0–3.0. Anticoagulation was continued for 3–6 months.

Catheter directed thrombolysis was performed only for lower limb ischaemia in patients who had thrombus only in the femoral or distal arteries and not in the aortoiliac segment. Infusion of recombinant tissue plasminogen activator alteplase (Actilyse, Boehringer Ingelheim, Ingelheim am Rhein, Germany) was carried out by an ipsilateral antegrade femoral approach. Two milligram alteplase was given as bolus dose which was followed by 0.015 mg per kg per hour infusion for 18–24 hours. Mechanical thrombectomy was not available during the study period. Patients who failed lysis or thrombectomy were taken up for open surgical bypass. Seventeen patients were managed conservatively with infusion of heparin while they were admitted and later were started on newer oral anticoagulants and vasodilators such as cilostazol after discharge.

For the present series, limb salvage was defined as the absence of major amputation or death within the study period. The other outcomes analysed were re-intervention rate, minor or major amputation and mortality.

## Results

A total of 67 limbs in 59 patients presented with COVID induced acute limb ischaemia. Eight patients had involvement of both lower limbs. Patients’ age varied from 37 to 91 years. Men (67.8%, n=40) were predominantly affected. Diabetes (66.1%, n=39) and hypertension (45.76%, n=27) were the most common co-morbid conditions. The other risk factors were previous vascular intervention (5.1%, n=3), coronary artery disease (11.86%, n=7), chronic kidney disease (1.7%, n=1) and smoking (5.1%, n=3).

Since this group of hospitals are tertiary care centres for vascular diseases majority of these patients were referred from other hospitals after completion of treatment for COVID-19. Demographics of the 59 patients treated are represented in **Table 1**. Average time taken from the onset of ischaemic symptom to intervention was 15 days. This is due to the delay in referral till the patient became COVID negative in most centres. During this time patients were given LMWH or UFH at the COVID designated hospitals.

**Table 1:** Table 1 Demographics of the patient population

Demographic	Value (n=59 patients)
Age (years)	59.26 (min–37, max–91)
Sex	M: 40 (67.8%), F: 19 (32.2%)
Diabetes	39 (66.1%)
Hypertension	27 (45.76%)
Coronary artery disease	7 (11.86%)
Smoking	
Current	1 (1.7%)
Former	2 (3.4%)
Previous vascular disease	3 (5.1%)
Previous cerebro-vascular accident	4 (6.78%)
Chronic kidney disease	1 (1.7%)
Time to presentation (avg)	15 days (min–1 day, max–28 days)
COVID status	
COVID RT-PCR positive	35 (59.32%)
O_2_ dependent	4 (6.78%)
COVID severity	
Asymptomatic	6 (10.17%)
Mild–moderate COVID	46 (78%)
Severe COVID	7 (11.86%)
Limb ischaemia as first presentation	6 (10.17%)
Associated venous thrombosis	5 (8.5%)
Associated PE	2 (3.4%)

avg: average; COVID: corona virus disease; RT-PCR: reverse transcription polymerase chain reaction; PE: pulmonary embolism; min: minimum; max: maximum

Six patients were asymptomatic for COVID, 46 had mild to moderate disease and 7 patients had severe COVID. Thirty-five patients were RT-PCR positive and the rest were diagnosed by pulmonary CT findings and positive COVID antibody test. None of the patients had received COVID vaccination at the time of the study. Four patients were oxygen dependent at the time of presentation.

There were 16 upper limbs (23.88%) and 51 lower limbs (76.12%) treated for acute limb ischaemia, the characteristics of which are presented in **Table 2**. Ischaemic pain started simultaneously with COVID symptoms in 6 patients. In 6 patients (10.17%), limb ischaemia was the only presenting symptom. Five patients developed ischaemic symptoms soon after discharge while the rest developed acute limb ischaemia during hospitalisation for COVID. All had received aspirin and therapeutic dose of LMWH during the hospitalisation. Patients who were referred from other COVID care centres early on in the pandemic had received only aspirin and did not receive anticoagulation after discharge.

**Table 2:** Table 2 Preoperative information and management details

Total number of ischaemic limbs	(n=67 Limbs)
Upper limb	16 (23.88%)
Lower limb	51 (76.12%)
Both lower limbs	8/59 (13.56%)
Side affected	RT: 36 (53.73%), LT: 31 (46.27%)
Severity of ischaemia (Rutherford)	
Class 1	27 (40.3%)
Class 2A	10 (14.93%)
Class 2B	18 (26.86%)
Class 3	12 (17.91%)
Site of thrombus	
Free-floating proximal thrombus (aortic/subclavian)	26 (38.8%)
Iliac artery	17 (25.37%)
Femoral artery	8 (13.56%)
Popliteal artery	23 (34.33%)
Isolated tibial	3 (4.5%)
Brachial artery	16 (23.88%)
Multi-segmental thrombotic involvement	45 (67.16%)
Procedures done	
Open thrombectomy- trans-femoral	14 (20.9%)
Open thrombectomy- trans-popliteal	10 (14.92%)
Both femoral and popliteal thrombectomy	5 (7.46%)
Primary bypass	2 (2.98%)
Trans-brachial embolectomy	8 (11.94%)
Lytic therapy	5 (7.5%)
Conservative management	17 (25.37%)

RT: right; LT: left

**Figure 1:**
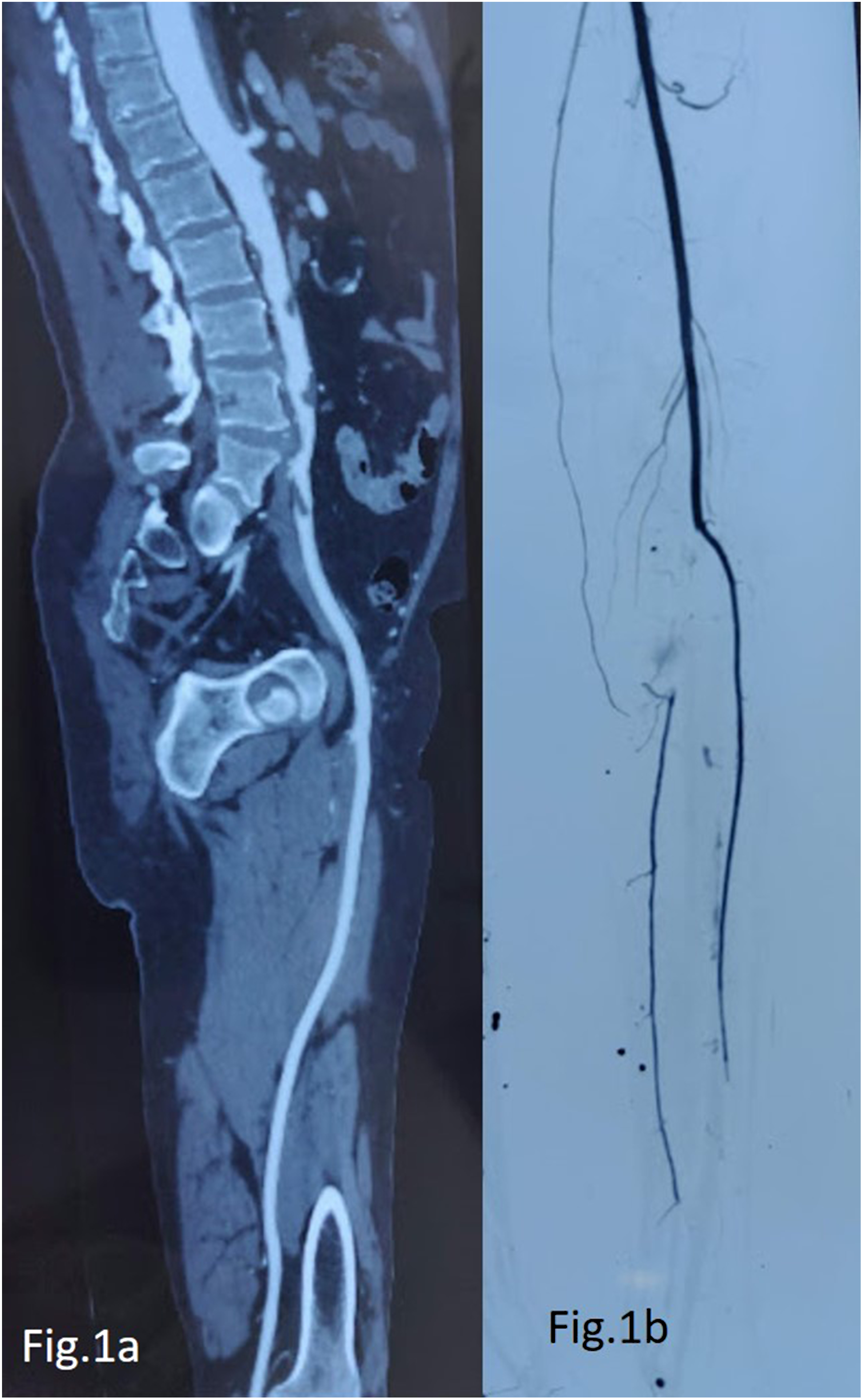
Fig. 1 (**a**) Computed tomography arteriogram showing non occlusive thrombus in the lower aorta and iliac artery at multiple sites. (**b**) Distal embolisation in the tibial vessels in the same patient.

**Figure 2:**
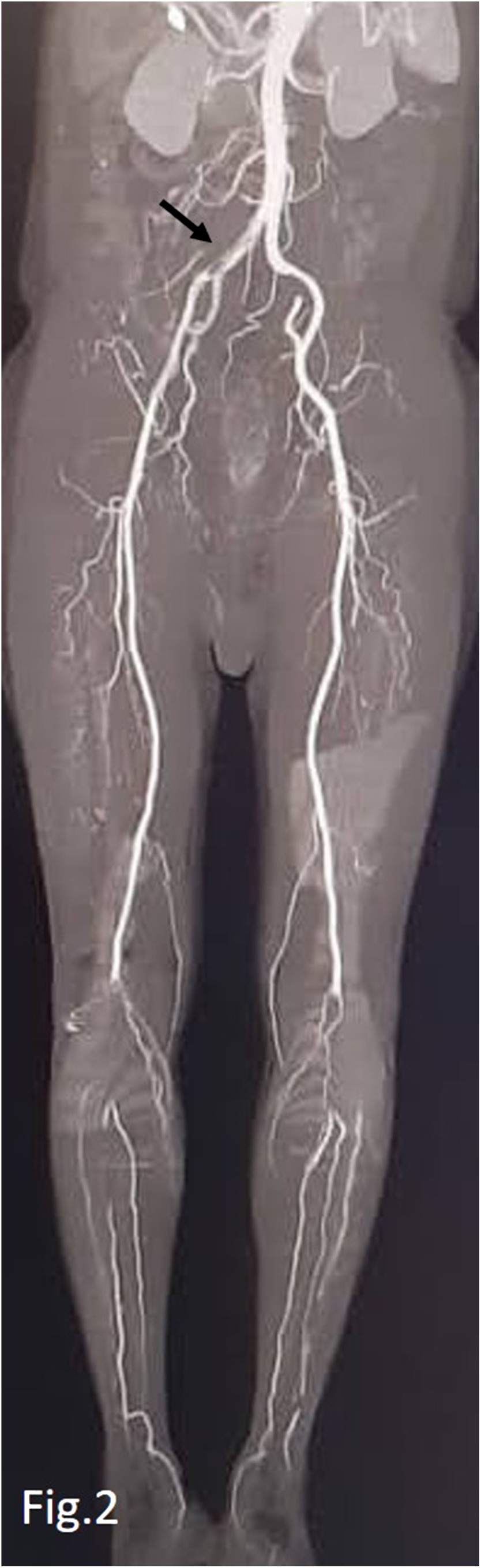
Fig. 2 Arteriogram showing floating thrombus in the distal aorta and right common iliac artery (arrow) with bilateral popliteal artery thrombotic occlusion.

CT aortogram was done in all patients. Non-occlusive, free-floating proximal thrombus was seen in 26 limbs (38.8%). The most common sites of thrombotic occlusions were iliac (n=17 limbs, 25.37%) and popliteal artery (n=23 limbs, 34.33%). Majority (45 limbs, 67.16%) of the patients showed multi segmental involvement (**Figs. 1** and **2**). Thirty-seven limbs had class 1 or 2A ischaemia (55.23%), 18 had class 2B (28.86%) and 12 had class 3 ischaemia (17.91%).

Two patients (3.4%) had incidental minor sub-segmental pulmonary embolism but were asymptomatic. Concomitant deep vein thrombosis was seen in five patients (8.5%). Thirteen patients (19.4%) had concomitant asymptomatic thrombus in minor vessels in the contralateral limb.

Of the 67 limbs, 39 (58.2%) were treated by open surgical revascularisation, 5 (7.5%) by catheter directed lysis, 17 (25%) were managed conservatively and 6 (9%) underwent primary amputation (**Table 3**). Of the limbs treated conservatively with anticoagulation, 10 (58.82%) were put on heparin infusion and 7 (41.18%) were managed as outpatients on newer oral anticoagulants or LMWH. Majority of these patients (n=14, 82.36%) had class 1 ischaemia and had thrombus in minor vessels causing digital or toe ischaemia and some who improved with anticoagulation or refused any intervention. The remaining had class 2B or 3 (n=3, 17.64%) ischaemia. These three patients with severe COVID on ventilator and higher grade of ischaemia had amputation or surgery deferred due to a poor prognosis. These patients succumbed to the COVID disease due to multi organ failure and passed away without intervention.

**Table 3:** Table 3 Comparison of various treatment modalities, results and complications (of 67 limbs)

	Open surgical revascularisation	Catheter directed lysis	Conservative	Primary amputation
Number (Limbs)	39	5	17	6
Class 1	11 (28.2%)	2 (40%)	14 (82.35%)	0
Class 2A	9 (23.08%)	1 (20%)	0	0
Class 2B	16 (41%)	1 (20%)	1 (5.88%)	0
Class 3	3 (7.69%)	1 (20%)	2 (11.76%)	6 (100%)
Limb salvage	35 (89.74%)	5 (100%)	14 (82.35%)	—
Re-intervention rate	4 (10.26%)	2 (40%)		—
Minor amputation	3 (7.69%)	2 (40%)	—	—
Major amputation	4 (10.26%)	0	0	—
Mortality	1 (2.56%)	0	3 (17.65%)	4 (66.67%)

Trans-brachial embolectomy was carried out in 8 limbs (11.94%), trans-femoral embolectomy in 14 (20.9%) and trans-popliteal in 10 (14.92%) for acute limb ischaemia. Both distal popliteal and femoral embolectomy was carried out in 5 limbs (7.46%). Two patients were taken up for primary bypass due to delay in presentation. Four patients (10.26%) developed immediate re-thrombosis after thrombectomy needing re-exploration. Re-thrombectomy was successful in 2. Two patients who had failed thrombectomy underwent successful reversed saphenous vein bypass. Four patients underwent major amputation after thrombectomy due to severe nature of tissue loss and superadded infection. One of them had iliofemoral vein thrombosis along with arterial thrombus while another patient who presented with class 3 ischaemia underwent femoral thrombectomy so that we could bring down the amputation level to below the knee.

Five patients (7.5%) underwent catheter directed thrombolysis (CDT). Thrombolysis failed in 2 limbs (40%) and both went on to have a distal bypass and limb salvage with minor amputations. Partial lysis was achieved in the other 3 and limb salvage could be achieved with continued anticoagulation. They had claudication but did not need any further intervention.

Six limbs (9%) underwent primary amputation for class 3 ischaemia following which 4 patients (66.67%) died due to severe nature of COVID. Two patients who had bilateral lower limb ischaemia had class 3 ischaemia in one leg and class 2B in the other. One limb was primarily amputated however the other limb in both could be salvaged with vascular intervention. Eight patients died during the study period due to severe lung involvement due to COVID and multi organ failure. However, 2 other patients with severe COVID underwent thrombectomy and limb salvage and recovered well.

A limb salvage rate of 80.6% (54/67 limbs) was achieved in these patients with a re-intervention rate of 13.6% (6/44 limbs). Major amputation rate was 14.92% (10/67) and mortality was 13.56% (8/59). All patients have been followed up for a minimum period of 6 months and none has shown any recurrence of ischaemia.

## Discussion

COVID, a viral respiratory illness caused by the SARS-CoV-2 predisposes patients to thrombosis in both the veins and arteries. This occurs due to an immune cross reaction with the viral components leading to endotheleitis, platelet activation and endothelial dysfunction.^4)^ This pathogenesis has been corroborated by the work on amputated specimens of patients with Rutherford class 3 ischaemia.^5)^ Endothelial injury with presence of inclusion bodies, widespread thrombosis and accompanying microangiopathy was demonstrated in multiple vascular beds of post mortem subjects.^1)^ This could explain thrombotic events even in asymptomatic or mild infection of COVID as well as those developing after discharge in these patients, involving multiple arterial segments.

Coagulopathy has emerged as one of the major factors contributing to complications and death following COVID-19 infection.^6)^ Following the first wave of the pandemic, most hospital policies changed to include prophylactic anticoagulation during admission and after discharge to address this complication. This helped reduce the incidence of ALI subsequently during the second wave. Despite anticoagulation, arterial thrombotic event has been reported in 3.7% of patients with severe COVID.^2)^ Being a tertiary referral service, majority of our patients were referred from other hospitals following signs of ALI. Some of them were referred late if their condition did not permit early transfer, which led to the late presentation in some of our patients. Ilonzo et al.^7)^ have reported arterial thrombosis as the first presenting symptom of COVID-19 infection in young patients with no comorbidities, as was seen in 6 patients in our study who had ALI as their presenting symptom. There was no correlation between severity of COVID and the severity of limb ischaemia (**Table 4**).

**Table 4:** Table 4 Comparison between severity of corona virus disease (COVID) and severity of limb ischaemia

Severity of COVID	Ischaemia Class 1 & 2A	Ischaemia Class 2B & 3	Total
Asymptomatic & mild	29 (55.76%)	23 (44.23%)	52
Moderate & severe	8 (53.33%)	7 (46.66%)	15
Total	37	30	67

Statistical analysis by Chi-square test, p-value=.867259 p<.05 was considered significant. No correlation between severity of COVID and limb ischaemia.

Many of our patients had developed arterial thrombosis after discharge. Majority of them had received only vitamins and supportive treatment and did not receive any anti-platelets or anticoagulants after discharge. Arterial thrombosis occurring in patients 2–3 weeks after recovery has been recently reported.^8)^ This is due to the continuation of the pro thrombotic state even after recovery. This raises the question of extended prophylaxis in these patients. This could be considered a sequela to COVID and a part of long COVID or post COVID syndrome now being identified in several case reports where the inflammatory process persists beyond 4 weeks.^9)^

Though all age groups have been seen to be affected in our study, majority belonged to 4th and 5th decades of life as was seen in the study by Indes et al.^10)^ Diabetes and hypertension were the most common comorbidities found in these patients. Iliac and popliteal artery were the most common sites of thrombotic occlusion. Twenty-six patients (38.8%) had a free-floating proximal thrombus in the aorto-iliac segment or proximal subclavian artery with distal artery involvement. Large vessel involvement with distal embolisation seems to be the most common presentation of peripheral ischaemia.^10)^ Every arterial bed has been reported to develop thrombus including the cerebral, coronary and the mesenteric vasculature.^6)^ Thirteen patients in our study had concomitant asymptomatic thrombus in other vascular beds. Multi-vessel involvement has been linked to a high incidence of mortality and morbidity.^6)^ Venous thrombosis is more common than arterial thrombosis in COVID patients.^11)^ Seven patients in our study were seen to have concomitant acute venous thrombosis along with arterial thrombosis. Presence of venous thrombosis did not correlate with either the severity of ischaemia or that of COVID. Whereas arterial thrombosis was more commonly associated with mortality than venous thrombosis. A high mortality of 13.56% (n=8) was observed in our study population.

Patients who are critically ill from COVID may be at too high a risk to undergo any surgical or endovascular attempts at limb salvage. Anticoagulation is essential while it is being determined whether the patient should undergo immediate or deferred surgical therapy. If the patient recovers from COVID, revascularisation can be considered if the limb is still salvageable, but amputation may be the only option if the limb progresses to non-viability. Therapeutic anticoagulation with unfractionated heparin has helped to tide over the emergency and limb salvage could be carried out after their lung condition improved. Heparin prevents propagation of the thrombus both proximally and distally and maintains patency of the collateral vessels. Most of the inflammatory thrombotic occlusion in COVID is segmental with an intact distal circulation in-spite of distal embolisation. Heparin helps to preserve the distal circulation till revascularisation becomes feasible.^12)^

The different approaches to acute limb ischaemia in COVID induced ischaemia have been outlined in several case reports. Open thrombectomy or lysis (systemic or catheter directed) have been used in patients with class 2A or 2B limb ischaemia. Catheter directed thrombolysis with mechanical thrombectomy devices have been found to be useful during the acute phase in active COVID as demonstrated by Aasen and Blecha.^13)^ Minimising procedure time and exposure seems to be hallmark of surgery in these patients with active COVID. Majority of our patients underwent open surgical thrombectomy (39 patients) as opposed to lysis in 5 patients. The common presentation in COVID being multi-segmental with associated arterio-arterial embolism, treatment of the proximal source of the thrombus is difficult when it involves the aorta as was seen in 18 of our patients.

Lysis and thrombectomy can be used to treat the distal embolic component leaving the proximal component to lyse naturally. This was demonstrated in the study by Muhammad et al.^14)^ Thrombus in these patients had a typical gelatinous consistency with areas of hard (white) and soft (black) components which appears different from the usual thrombotic specimens seen in non-COVID cases. This could be the reason why thrombolysis was not completely successful in these patients. Five patients were taken up for CDT and was partially successful in 3. The other two required a bypass to save the leg. Failure of catheter directed thrombolysis alone to remove these inflammatory hard thrombi has been reported by Vacirca et al.^15)^ Completion angiography in these patients showed multiple small vessel thrombi causing occlusion in the distal circulation. This can be the cause for early recurrent thrombosis in these patients. Pharmaco-mechanical thrombectomy seems to hold some promise in COVID induced arterial thrombosis during the acute phase as shown by Aasen and Blecha.^13)^ We did not have access to mechanical thrombectomy in our centres at that time. To improve the results after open thrombectomy, we routinely used intra-operative thrombolysis with urokinase in the distal arterial tree and continuous infusion of UFH in the post-operative period which has improved these results. This has been reported in other studies.^3,12)^ No bleeding related complication were seen post-operatively.

In spite of a delay, successful thrombectomy even 20 days after the onset of symptoms was carried out in 13 of the 39 patients. Four patients developed recurrence of thrombosis and were re-explored of which two patients required a distal bypass. Dense periarterial inflammatory adhesion and brownish staining of intima were seen in these patients who had re-thrombosis indicating transmural inflammation. This inflamed intima and residual adherent thrombus predisposes these patients to re-thrombosis. It is better to do bypass to the patent distal segment in these patients. We could achieve a limb salvage rate of 80.6% (54/67 limbs) which is much better than what has been reported.^3)^ Bellosta et al.^3)^ reported disappointingly low results after open revascularisation in patients with COVID but our results have been comparable to limb salvage achieved in ALI after non COVID causes.^16)^

A definitive recommendation cannot be provided because of the lack of consistent published data. Moreover, to date, we lack large prospective cohorts and the existing evidence is derived primarily from small and retrospective case series.

## Conclusion

Arterial thrombosis after COVID is not uncommon. Optimal use of anticoagulation during treatment of COVID and continuing prophylaxis post discharge in high-risk patients suffering from COVID helps to reduce the incidence of ALI. Most patients with COVID induced ALI have thrombus in the proximal artery with distal embolisation. Open thrombectomy can be safely attempted even in late presentation. Re-thrombosis which is high can be reduced by routine use of thrombolytics into the distal arterial tree and unfractionated heparin infusion in the post operative period. Re-thrombosis can be predicted if there is dense peri arterial adhesions and brown staining of intima. If thrombectomy fails, bypass to the patent distal arterial segment can be undertaken safely in these patients. Similar rates of limb salvage as in non-COVID ALI can be achieved.
